# Altered GABAergic markers, increased binocularity and reduced plasticity in the visual cortex of Engrailed-2 knockout mice

**DOI:** 10.3389/fncel.2014.00163

**Published:** 2014-06-17

**Authors:** Manuela Allegra, Sacha Genovesi, Marika Maggia, Maria C. Cenni, Giulia Zunino, Paola Sgadò, Matteo Caleo, Yuri Bozzi

**Affiliations:** ^1^Neuroscience Institute, National Research Council (CNR)Pisa, Italy; ^2^Laboratory of Neurobiology, Scuola Normale SuperiorePisa, Italy; ^3^Laboratory of Molecular Neuropathology, Centre for Integrative Biology, University of TrentoMattarello, Trento, Italy

**Keywords:** plasticity, inhibition, monocular deprivation, critical period, neurodevelopmental disorder

## Abstract

The maturation of the GABAergic system is a crucial determinant of cortical development during early postnatal life, when sensory circuits undergo a process of activity-dependent refinement. An altered excitatory/inhibitory balance has been proposed as a possible pathogenic mechanism of autism spectrum disorders (ASD). The homeobox-containing transcription factor Engrailed-2 (*En2*) has been associated to ASD, and *En2* knockout (*En2*^−/−^) mice show ASD-like features accompanied by a partial loss of cortical GABAergic interneurons. Here we studied GABAergic markers and cortical function in *En2*^−/−^ mice, by exploiting the well-known anatomical and functional features of the mouse visual system. *En2* is expressed in the visual cortex at postnatal day 30 and during adulthood. When compared to age-matched *En2*^+/+^ controls, *En2*^−/−^ mice showed an increased number of parvalbumin (PV^+^), somatostatin (SOM^+^), and neuropeptide Y (NPY^+^) positive interneurons in the visual cortex at P30, and a decreased number of SOM^+^ and NPY^+^ interneurons in the adult. At both ages, the differences in distinct interneuron populations observed between *En2*^+/+^ and *En2*^−/−^ mice were layer-specific. Adult *En2*^−/−^ mice displayed a normal eye-specific segregation in the retino-geniculate pathway, and *in vivo* electrophysiological recordings showed a normal development of basic functional properties (acuity, response latency, receptive field size) of the *En2*^−/−^ primary visual cortex. However, a significant increase of binocularity was found in P30 and adult *En2*^−/−^ mice, as compared to age-matched controls. Differently from what observed in *En2*^+/+^ mice, the *En2*^−/−^ primary visual cortex did not respond to a brief monocular deprivation performed between P26 and P29, during the so-called “critical period.” These data suggest that altered GABAergic circuits impact baseline binocularity and plasticity in *En2*^−/−^ mice, while leaving other visual functional properties unaffected.

## Introduction

Mice lacking the homeobox-containing transcription factor Engrailed-2 (*En2*) represent a good animal model to study the neurodevelopmental basis of autism spectrum disorders (ASD). Genome-wide association studies indicated *En2* as a candidate gene for ASD (Benayed et al., 2009). *En2* knockout (*En2*^−/−^) mice display cerebellar hypoplasia (Joyner et al., 1991), reduced number of Purkinje cells (Kuemerle et al., 1997) and ASD-like behaviors (Cheh et al., 2006; Brielmaier et al., 2012). In particular *En2*^−/−^ animals display very significant deficits in reciprocal social interactions as juvenile and adults (Brielmaier et al., 2012). In addition to the ASD-like phenotype, *En2*^−/−^ mice display an increased susceptibility to seizures (Tripathi et al., 2009), which is often observed in ASD patients (Gilby and O'Brien, 2013). Recent studies from our laboratory showed that *En2* is expressed in the adult forebrain (Tripathi et al., 2009), and that *En2*^−/−^ mice present a significant loss of parvalbumin (PV^+^), somatostatin (SOM^+^), and neuropeptide Y (NPY^+^) positive GABAergic interneurons in the adult hippocampus and somatosensory cortex (Sgadò et al., 2013a).

Abnormalities in GABAergic circuitry have been reported in the forebrain of ASD mouse models (Gogolla et al., 2009; Provenzano et al., 2012). Altered development, connectivity and/or function of GABAergic interneurons might result in an unbalanced ratio of excitation/inhibition and might represent the anatomical substrate of an immature structure and function of the cerebral cortex, as postulated to occur in the autistic brain (Rubenstein and Merzenich, 2003).

The visual cortex has been used as a model system to test the physiological consequences of an altered development of the GABAergic system. As an assay of the potential for plasticity, many groups employed the paradigm of monocular eyelid suture (monocular deprivation, MD). It is well-known that reducing visual input from one eye during development leads to a loss of physiological responses to that eye, and to a change in the eye preference of cortical neurons referred to as ocular dominance (OD) shift (Hubel and Wiesel, 1963). OD plasticity is markedly pronounced during a specific developmental time window termed the “critical period” (Levelt and Hübener, 2012) but can still be induced to a certain degree during early adulthood in mice (Sawtell et al., 2003; Hofer et al., 2006; Lehmann and Löwel, 2008; Sato and Stryker, 2008). A clear OD shift can be observed in juvenile (P26-P32) mice with 3 days of MD, while adults require longer deprivation periods for the OD shift to become significant (Sawtell et al., 2003; Lehmann and Löwel, 2008; Sato and Stryker, 2008; Tropea et al., 2009a).

Previous work examined the role of GABAergic inhibition in the control of OD plasticity, showing that the proper maturation of the GABAergic system is a crucial determinant of cortical plasticity during postnatal development. Specifically the levels of intracortical inhibition are crucial for initiating the critical period for OD plasticity (Hensch et al., 1998; Huang et al., 1999). Several additional mechanisms are involved in the closure of the critical period, including maturation of extracellular matrix molecules and myelin-associated growth inhibitors, and a reduction of CREB-mediated gene transcription (Pizzorusso et al., 2002; Pham et al., 2004; McGee et al., 2005). The level of inhibition may also control critical period closure, as pharmacological reduction of GABAergic neurotransmission in the visual cortex facilitates adult OD plasticity (Harauzov et al., 2010; Greifzu et al., 2014). Susceptibility to MD has recently been used to assess plasticity of cortical networks in models of mental retardation such as *Fmr1* (Doelen et al., 2007) and *MeCP2* (Tropea et al., 2009b) mutant mice, which are known to present anatomical and functional deficits of cortical GABAergic interneurons (Centonze et al., 2008; Curia et al., 2009; Chao et al., 2010).

In this study, we describe alterations of GABAergic interneurons in the visual cortex of *En2*^−/−^ mice, and its impact on experience-dependent cortical plasticity both during the critical period and in the adult age.

## Materials and methods

### Animals

Experiments were performed in compliance to European Communities Council Directive of 24 November 1986 (86/609/EEC), and approved by the Italian Ministry of Health. Animals were housed in a 12 h light/dark cycle with food and water available *ad libitum*, and all efforts were made to minimize animal suffering during the experiments. The original *En2* mutants (mixed 129Sv × C57BL/6 genetic background; Joyner et al., 1991) were crossed at least five times into a C57BL/6 background. Heterozygous mating (*En2*^+/−^ × *En2*^+/−^) were used to generate the *En2*^+/+^ and *En2*^−/−^ littermates used in this study. Genotyping was performed by PCR as previously described (Sgadò et al., 2013a). For all the experiments, *En2*^+/+^ and *En2*^−/−^ age-matched littermates at postnatal (P) days 26–30 or adult age (age range: P90–P120) were used. Animals of both sexes were used in all experiments, since our previous studies did not reveal any sex-related difference in GABAergic neurons anatomy in *En2*^−/−^ mice (Tripathi et al., 2009; Sgadò et al., 2013a). For immunohistochemistry experiments, only female animals were used. Male and female animals in approximately equal proportions were instead used for electrophysiology experiments. Previous behavioral studies showed that male and female *En2*^−/−^ mice do not show significant differences in visually-driven tasks (Brielmaier et al., 2012).

### Quantitative RT-PCR

Total RNAs were extracted by Trizol reagent (Invitrogen) from primary visual cortices explanted from P30 and adult *En2*^+/+^ and *En2*^−/−^ mice (*n* = 4 per genotype and age group). DNAse-treated RNAs were purified by RNA extraction RNAeasy Kit (Qiagen), and pooled. cDNA was synthesized from pooled RNAs (3 μg) by SuperScript VILO cDNA Synthesis Kit (Invitrogen). Quantitative reverse-transcription PCR (RT-PCR) was performed in a C1000 Thermal Cycler (BioRad) with real-time detection of fluorescence, using the KAPA SYBR FAST Master Mix reagent (KAPA Biosystems, USA). Mouse mitochondrial ribosomal protein L41 (Mrpl41) was used as a standard for quantification. Primers (Sigma Genosys, UK) sequences are reported in Table 1. Ratios of comparative concentrations of each mRNA with respect to L41 mRNA were then calculated and plotted as the average of three to four independent reactions with technical replicates obtained from each RNA pool. Expression analyses were performed using the CFX3 Manager (BioRad) software.

**Table 1:** **Primers used for quantitative RT-PCR experiments**.

**Gene**	**Genbank #**	**Forward primer (5′–3′)**	**Reverse primer (5′–3′)**
*En2*	NM_010134.3	ACTGCACGCGCTATTCTG	ACCTGTTGGTCTGAAACTCAG
CALB	NM_009788	AGATCTGGCTTCATTTCGACG	TTCATTTCCGGTGATAGCTCC
GAD67	NM_008077	TCCAAGAACCTGCTTTCCTG	GAGTATGTCTACCACTTCCAGC
NPY	NM_023456	TCACAGAGGCACCCAGAG	AGAGATAGAGCGAGGGTCAG
PV	NM_013645	TGCTCATCCAAGTTGCAGG	GCCACTTTTGTCTTTGTCCAG
SOM	NM_009215	AGGACGAGATGAGGCTGG	CAGGAGTTAAGGAAGAGATATGGG
vGAT	NM_009508	TCACGACAAACCCAAGATCAC	GTCTTCGTTCTCCTCGTACAG
vGLUT1	NM_182993	CACAGAAAGCCCAGTTCAAC	CATGTTTAGGGTGGAGGTAGC

See text for abbreviations.

### Immunohistochemistry, cell counts, and morphometric analysis

Brains from P30 and adult *En2*^+/+^ and *En2*^−/−^ mice (*n* = 3 per genotype and age group) were used for immunohistochemical characterization of GABAergic cells. Brains were fixed by transcardial perfusion with 4% paraformaldehyde followed by 1 h post-fixation, and coronal sections (40 μm thickness) were cut by a vibratome (Leica). Serial sections at level of the visual cortex were incubated overnight with appropriate antibodies as follows: anti-parvalbumin (PV) mouse monoclonal (Sigma; 1:2000 dilution); anti-somatostatin (SOM) rabbit polyclonal (Peninsula-Bachem; 1:2000 dilution); anti-neuropeptide Y (NPY) rabbit polyclonal (Peninsula-Bachem, UK; 1:2000 dilution); anti-NeuN mouse monoclonal (Millipore; 1:500 dilution). Signals were revealed using appropriate secondary antibodies and fluorofores as described (Sgadò et al., 2013a).

Three to 5 sections at the level of the primary visual cortex were analyzed per animal (3 mice per age and genotype). Primary visual cortex (V1) was identified according to the Allen Brain Atlas (http://www.brain-map.org/). Multiple images from each section were acquired at 20× objective magnification using a Zeiss Observer Z1 microscope, and then assembled using the MosaiX tool of the Zeiss AxioVision v4.8.1 software to reconstruct the entire section. Light intensity and microscope settings were optimized initially and then kept constant to maintain the same exposure through the single microphotographs and sections overall. Cell counts were then performed on tiff-converted mosaic images using Adobe Photoshop and ImageJ (http://rsb.info.nih.gov/ij/) softwares. Antibody-stained cells were separately counted in superficial (II–III) and deep (V–VI) layers of primary visual cortex over 2 to 3 counting boxes of 200 × 600 μ m each. Cell densities were expressed as the number of labeled cells per counting window (200 × 600 μ m). To establish a consistent guideline for counting individual cells, only cells larger than 5 μm with a clearly visible nucleus were counted. Signals smaller than 5 μm were excluded to avoid counting neurites, nerve terminals, and false signals. For morphometric analysis, bright-field images of the primary visual cortex stained with a NeuN antibody were acquired at 20× primary magnification using the Zeiss Observer Z1 microscope and merged by the MosaiX tool. Morphometric analysis of cortical layers was performed measuring layer thickness by ImageJ software on 4–6 NeuN-stained sections per animal (Sgadò et al., 2013a). Layers thickness was expressed as the percentage of total cortical thickness. All counts and measurements were performed by an experimenter blind of genotypes.

### Monocular deprivation

Monocular eyelid suture was performed under isoflurane anesthesia as described (Pinto et al., 2009; Restani et al., 2009). Animals were checked daily to make sure that the lid suture remained intact. All animals were recorded 3 days after MD. This protocol of brief MD was chosen since it produces robusts OD shifts during the critical period but not in adulthood (Sawtell et al., 2003; Lehmann and Löwel, 2008; Sato and Stryker, 2008). OD recordings were performed in both hemispheres (contralateral and ipsilateral to the deprived eye). For assessing MD effects, we used the following number of animals: P28 (contralateral hemisphere), *n* = 5 for *En2*^+/+^ and *n* = 4 for *En2*^−/−^ mice; P28 (ipsilateral hemisphere), *n* = 5 for *En2*^+/+^ and *n* = 5 for *En2*^−/−^ animals; adult age (contralateral hemisphere), *n* = 4 for both genotypes.

### *In vivo* electrophysiology

Mice were anesthetized with Hypnorm/Hypnovel (in water; 0.3 mL/20 g; VetaPharma, UK) and placed in a stereotaxic apparatus. Additional doses of anesthetic (0.05 mL/100 g) were given to keep the level of anesthesia stable. A portion of the skull overlying the binocular visual cortex was drilled on one side. A glass micropipette (tip diameter, 4 μm; 1 MΩ) filled with NaCl (3 M) or a tungsten electrode (1 MΩ; FHC, USA) was mounted on a three-axis motorized micromanipulator and inserted into the binocular portion of the visual cortex (approximately 2.9 mm lateral from midline and in correspondence with lambda in P28 mice and 3.2 mm lateral from midline and in correspondence with lambda in adult animals). VEPs were recorded from 3 to 4 penetrations/animal and the electrode was positioned at 100 and 400 μm depth within the cortex. Electrical signals were amplified (10,000 fold), band-pass filtered (0.3–100 Hz), digitized and averaged in synchrony with the stimulus contrast reversal. Analysis of the amplitude of VEP responses was performed blind to animal genotype. Visual stimuli were gratings of various spatial frequencies and contrast generated by a VSG2/5 card (Cambridge Research Systems, Rochester, UK) on a display (Sony Multiscan G500; mean luminance, 15 cd/m2) that was positioned 20-30 cm in front of the mouse eyes to include the central visual field (110 × 85° of visual angle).

VEP recordings in steady-state mode were used to measure spatial resolution. The visual response was measured as the amplitude (μV) of the second harmonic of the stimulation frequency (4 Hz), calculated after Fourier analysis of the signal. Visual acuity was assessed after presentation of gratings of variable spatial frequencies (90% contrast). Visual acuity was determined as the lowest spatial frequency that evoked a VEP response greater than the mean value of the noise.

Transient VEPs were recorded in response to the abrupt contrast reversal (1 Hz) of a square-wave grating (spatial frequency 0.06 c/deg, contrast 90%). At least 50 responses were averaged. For each animal, we calculated a C/I ratio, i.e., the ratio of VEP amplitudes recorded by stimulating the contralateral and ipsilateral eye. The degree of OD plasticity in each monocularly-deprived (MD) animal was quantified by dividing the mean value of the C/I ratio in non-MD mice of the same group and the C/I recorded in that specific animal, and multiplying this ratio by 100. Therefore, the index is around 100 when MD has no effect and progressively increases with the magnitude of the OD shift. For VEP recordings of basic physiological parameters we used the following number of animals: P28, *n* = 6 for both *En2*^+/+^ and *En2*^−/−^; adult age, *n* = 4 for both genotypes.

For extracellular recordings of spiking activity the visual stimulus consisted of a computer-generated bar (contrast, 90%; thickness, 3°; speed, 25°/s). Spontaneous activity and peak response were determined from peristimulus time histograms (PSTHs) of the cell response to the stimulus, averaged over 20 consecutive stimulations as described previously (Restani et al., 2009; Cerri et al., 2010). Action potentials were discriminated from background by a voltage threshold, that was set as 4.5 times the standard deviation of noise as described (Restani et al., 2009). OD was evaluated according to the methods of Hubel and Wiesel (1962). Neurons in OD class 1 were driven only by stimulation of the contralateral eye. Neurons in OD class 2/3 were binocular and preferentially driven by the contralateral eye. Neurons in OD class 4 were equally driven by the two eyes. Neurons in OD class 5/6 were binocular and preferentially driven by the ipsilateral eye. Neurons in OD class 7 were driven only by the ipsilateral eye. To obtain a finer and statistically more robust comparison of OD distributions, we computed the normalized OD score of single neurons (Rittenhouse et al., 1999) and plotted the cumulative distribution for each experimental group. OD score was computed on cells with complete PSTH analysis of peak and baseline spiking activity after closure of either eye. OD score was defined as (peak_ipsi_ − baseline_ipsi_) − (peak_contra_ − baseline_contra_) / (peak_ipsi_ − baseline_ipsi_) + (peak_contra_ − baseline_contra_). For extracellular recordings of spiking activity we used the following number of animals: *En2*^+/+^ P28, *n* = 5 (93 cells); *En2*^−/−^ P28, *n* = 5 (86 cells); *En2*^+/+^ adult, *n* = 5 (110 cells); *En2*^−/−^ adult, *n* = 5 (103 cells).

### Labeling and analysis of retino-geniculate axons

Labeling of retino-geniculate axons was performed as previously described (Menna et al., 2003). In brief, adult mice (*n* = 5 for *En2*^+/+^ and *n* = 3 for *En2*^−/−^) received an intravitreal injection of Cholera Toxin B subunit (CTB) conjugated with Alexa Fluor 488 (10 mg/mL, Invitrogen) in the left eye and CTB-Alexa Fluor 594 (10 mg/mL, Invitrogen) in the right eye 2 days before perfusion. Animals were perfused transcardially with 4% paraformaldehyde in 0.1 M phosphate buffer. Brains were post fixed for 2 h at 4°C, and cryoprotected in 30% sucrose in 0.1 M PB at 4°C. Coronal sections (50 μm thick) were cut on a freezing microtome and collected in a serial order through the entire thalamus. CTB labels were examined with a Leica Confocal microscope using a 10× air objective. For each animal, we acquired the five largest sections through the middle of the dorsal lateral geniculate nucleus (dLGN), where the two eye-specific domains appear better segregated (Rossi et al., 2001; Stellwagen and Shatz, 2002; Menna et al., 2003). For each section, confocal series of a step size of 2 μm were obtained throughout the whole section thickness (50 μm), and collapsed as an average. The collected images of the dLGN were used to analyze the areas occupied by the ipsilateral and the contralateral afferents with MetaMorph software (Molecular Devices, Sunnyvale, CA). In each section, the outline of the ipsilateral and contralateral projections and of the entire dLGN were drawn and their area was measured. For each animal, the profile of ipsilateral and contralateral dLGN projections were drawn on the computer screen excluding the ventral LGN and extrageniculate optic tract. The relative areas occupied by the ipsilateral and contralateral projections were calculated by dividing the average of the five ipsilateral or contralateral areas by the average of the five total dLGN areas. To determine the extent of overlap between ipsilateral and contralateral projections to the same dLGN, the ipsilateral and contralateral areas were measured, and their sum was subtracted from the total dLGN area and expressed as a percentage of it.

### Statistical analysis

Statistical analysis was performed with SigmaPlot 11.0 and Prism 6 (GraphPad) softwares. Data normally distributed were summarized by mean ± s.e.m., whereas data non-normally distributed were summarized with percentiles and a box chart. Pairwise comparisons of quantitative phenotypes between *En2*^+/+^ and *En2*^−/−^ mice were assessed by a two-tailed Student's *t*-test. When more than two groups/factors were analyzed, Two-Way ANOVA followed by appropriate *post hoc* test (Tukey's or multiple *t*-test) or One-Way ANOVA on ranks with Dunn's *post hoc* test were used for data normally or not normally distributed, respectively. Level of significance was set at *p* < 0.05.

## Results

### Expression of GABAergic and glutamatergic markers in the visual cortex of *En2*^−/−^ mice

Our previous studies showed that *En2* is expressed in the postnatal (P30) and adult mouse hippocampus and somatosensory cortex, and that *En2* inactivation results in the partial loss of GABAergic interneurons in these brain regions (Tripathi et al., 2009; Sgadò et al., 2013a).

**Figure 1:**
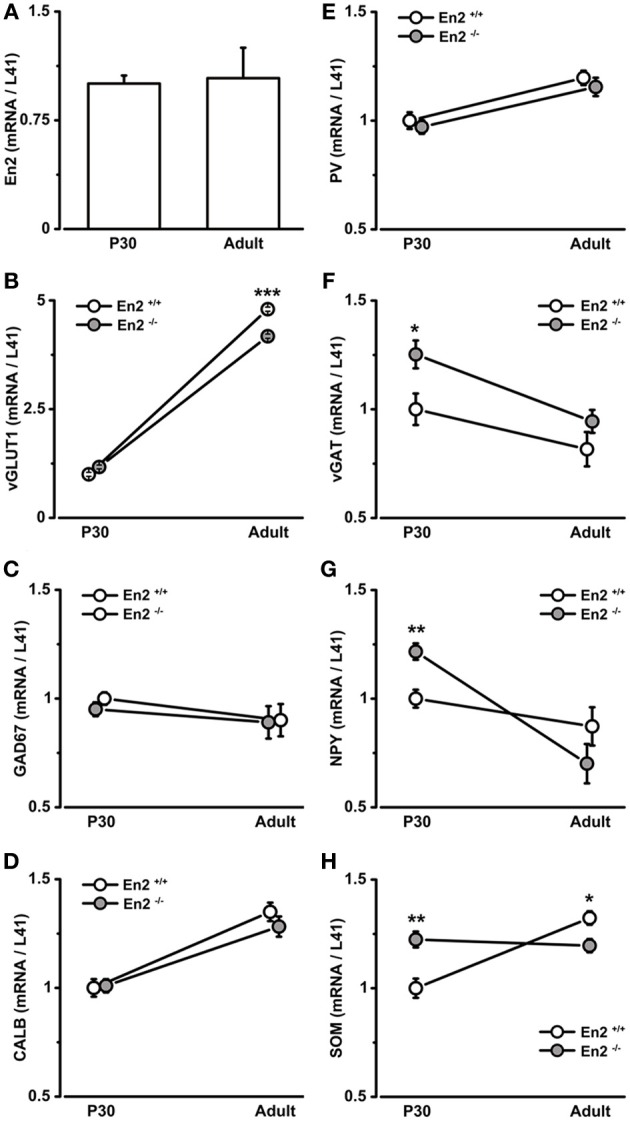
**mRNA expression of *En2* and glutamatergic/GABAergic markers in the juvenile and adult visual cortex of *En2*^+/+^ and *En2*^−/−^ mice. (A–H)** Relative mRNA expression level as obtained by quantitative RT-PCR performed on the visual cortex of P30 and adult *En2*^+/+^ (white) and *En2*^−/−^ (gray) mice. (**A**, *En2*; **B**, vGLUT; **C**, GAD67; **D**, CALB; **E**, PV; **F**, vGAT; **G**, NPY and **H**, SOM). Values are expressed as each marker/L41 comparative quantitation ratios (mean ± s.e.m.). vGAT, NPY, and SOM mRNA levels were significantly higher in *En2*^−/−^ mice at P30, whereas vGLT and SOM mRNA levels were higher in adult *En2*^+/+^ mice (see text for details). Statistical significance: ^*^p < 0.05, ^**^p < 0.01, ^***^p < 0.001 (*En2*^+/+^ vs. *En2*^−/−^). Abbreviations as in the text.

Quantitative RT-PCR confirmed that *En2* mRNA is expressed in both P30 and adult (P90–P120) mouse primary visual cortex, with no differences between the two ages (Figure 1A). We next used quantitative RT-PCR to evaluate the expression of glutamatergic and GABAergic markers in the visual cortex of *En2*^+/+^ and *En2*^−/−^ mice at P30 and adult age. The age of P30 was chosen as it is generally considered the peak of the normal critical period for OD plasticity in mice (Gordon and Stryker, 1996).

Vesicular glutamate transporter 1 (vGLUT1) was used as a marker of glutamatergic neurons, whereas glutamic acid decarboxylase (67 kDa isoform; GAD67) and the vesicular GABA transporter (vGAT) were used as markers of GABAergic interneurons. Cortical interneuron subpopulations were identified by the expression of specific intracellular markers, such as PV, SOM, and NPY (Mátyás et al., 2004; Ascoli et al., 2008; Jinno and Kosaka, 2010; Rudy et al., 2011); calbindin (CALB) instead labels both glutamatergic and GABAergic neurons (Celio, 1990).

vGLUT1 mRNA expression significantly increased from P30 to the adult age in both genotypes (3.8 fold in *En2*^+/+^, 2.6 fold in *En2*^−/−^; Two-Way ANOVA followed by Tukey test, P30 vs. adult, *p* < 0.001, *n* = 4 per genotype and age group) (Figure 1B), but was significantly lower in *En2*^−/−^ adult mice, as compared to age-matched *En2*^+/+^ controls (–13% in *En2*^−/−^; Two-Way ANOVA followed by Tukey test, *p* < 0.001, *n* = 4 per genotype) (Figure 1B). No differences were found in GAD67 mRNA levels between genotypes at both time points (Figure 1C; Two-Way ANOVA followed by Tukey test, *p* > 0.05 for all comparisons, *n* = 4 per genotype and age group). Accordingly, *in situ* hybridization showed no difference in GAD67-positive cell density in the adult visual cortex between the *En2*^+/+^ and *En2*^−/−^ mice (data not shown). CALB and PV mRNAs showed a statistically significant increase from P30 to the adult age in both *En2*^+/+^ and *En2*^−/−^ mice (CALB, +35% in adult *En2*^+/+^ and +27% in adult *En2*^−/−^; PV, +20% in adults in both genotyopes; Two-Way ANOVA followed by Tukey test; *p* < 0.05 for all comparisons, *n* = 4 per genotype and age group) (Figures 1D,E), while no significant differences were found between genotypes at P30 or adult age (Figures 1D,E; Two-Way ANOVA followed by Tukey test; *p* > 0.05 for all comparisons, *n* = 4 per genotype and age group).

A different expression profile was observed for vGAT, NPY, and SOM mRNAs. At P30, both vGAT and NPY mRNA levels were significantly higher in *En2*^−/−^ mice as compared to *En2*^+/+^ controls (vGAT, +25%; NPY, +22%; Two-Way ANOVA followed by multiple *t*-test, *p* = 0.04 for vGAT and *p* = 0.008 for NPY; *n* = 4 per genotype) (Figures 1F,G). They significantly decreased in *En2*^−/−^ mice from P30 to the adult age (vGAT, −25%; NPY, −42%; Two-Way ANOVA followed by Tukey test, *p* = 0.033 for vGAT and *p* < 0.001 for NPY; *n* = 4 per genotype and age group) and did not differ between the two genotypes in adult animals (Figures 1F,G). Finally, SOM mRNA levels in *En2*^−/−^ mice were higher (+22%) at P30 and lower (−10%) at adult age, as compared to *En2*^+/+^ controls (P30, Two-Way ANOVA followed by Tukey test, *p* = 0.006; adult, Two-Way ANOVA followed by multiple *t*-test, *p* = 0.04; *n* = 4 per genotype and age group) (Figure 1H). Table 2 summarizes the significant differences in glutamatergic and GABAergic markers observed between the *En2*^+/+^ and *En2*^−/−^ visual cortices at P30 and adult age. These results indicate an increase in several inhibitory markers in the visual cortex of *En2*^−/−^ mice at P30.

**Table 2:** **Expression of glutamatergic and GABAergic markers in the visual cortex: significant differences between *En2*^−/−^ and *En2*^+/+^ mice**.

**Markers/age**	**% difference (*En2*^−/−^ vs. *En2*^+/+^)**	***p*-value**
**P30**		
vGAT mRNA	+25	0.040
NPY mRNA	+22	0.008
SOM mRNA	+22	0.006
NPY^+^ cells (layers II–III)	+21	0.017
SOM^+^ cells (layers V–VI)	+14	0.020
PV^+^ cells (layers V–VI)	+20	0.030
**ADULT**		
vGLUT mRNA	−13	<0.001
SOM mRNA	−10	0.040
SOM^+^ cells (layers II–III)	−25	0.024
NPY+ cells (layers V–VI)	−20	0.040

See text for abbreviations.

### Increased density of SOM^+^, NPY^+^, and PV^+^ cells in the visual cortex of *En2*^−/−^ juvenile mice

**Figure 2:**
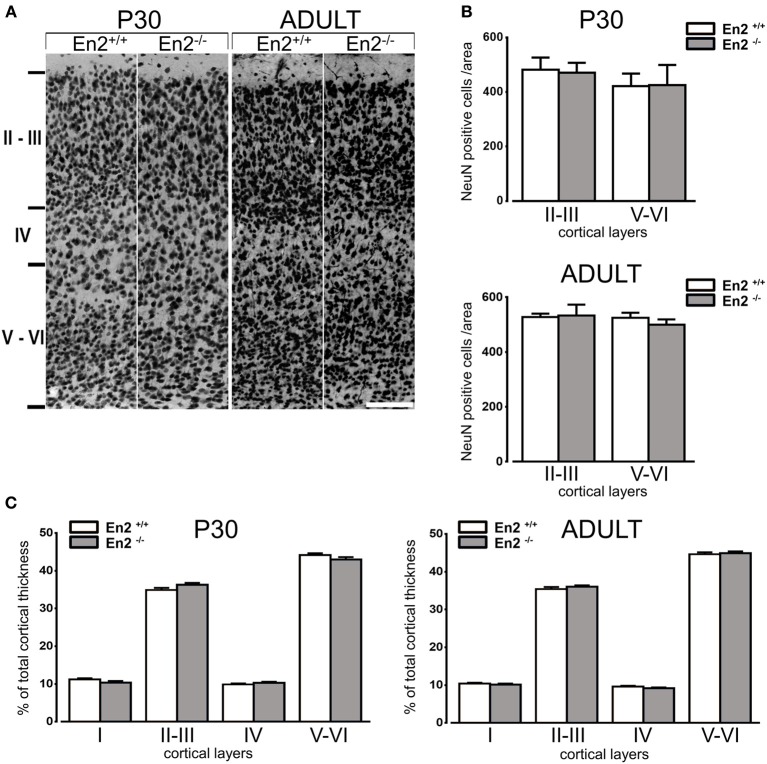
**Normal neuronal density and layering in the primary visual cortex of P30 and adult *En2*^−/−^ mice. (A)** Representative NeuN immunostaining of the primary visual cortex from P30 and adult *En2*^+/+^ (white) and *En2*^−/−^ (gray) mice. **(B)** Quantification of NeuN immunohistochemistry experiments. Cell densities in layers II–III and V–VI are plotted as the mean number (± s.e.m.) of cell per area (see Materials and Methods). **(C)** Morphometric analysis of cortical layers in P30 and adult *En2*^+/+^ and *En2*^−/−^. Layer thickness is plotted as % of total cortical thickness. Scale bar = 100 μm.

The expression profile of GABAergic marker mRNAs in the visual cortex prompted us to investigate the cell density of GABAergic interneurons in the primary visual cortex of *En2*^+/+^ and *En2*^−/−^ mice both during the critical period and in adulthood. We first verified that total neuron numbers and layering of the visual cortex were comparable between *En2*^+/+^ and *En2*^−/−^ mice both at P30 and in adulthood. Counts of NeuN-positive cells (Figure 2A) did not reveal any significant difference between *En2*^+/+^ and *En2*^−/−^ mice at both ages analyzed (*p* > 0.05, Two-Way ANOVA followed by Holm-Sidak test; *n* = 3 per genotype) (Figure 2B). NeuN immunohistochemistry also revealed no gross layering defects in the primary visual cortex of both P30 and adult *En2*^−/−^ mice, as compared to *En2*^+/+^ littermates (Figure 2C). Morphometric analysis showed that at both ages analyzed, the total thickness of the primary visual cortex (P30 *En2*^+/+^, 1033 ± 10.95 μm; P30 *En2*^−/−^, 1042 ± 14.76 μm; adult *En2*^+/+^, 1018 ± 15.20 μm; adult *En2*^−/−^, 985.2 ± 8.95 μm) as well as the thickness of each cortical layer (Figure 2C) did not differ between between genotypes (Two-Way ANOVA followed by Tukey test, *p* > 0.05; *n* = 3 per genotype).

**Figure 3:**
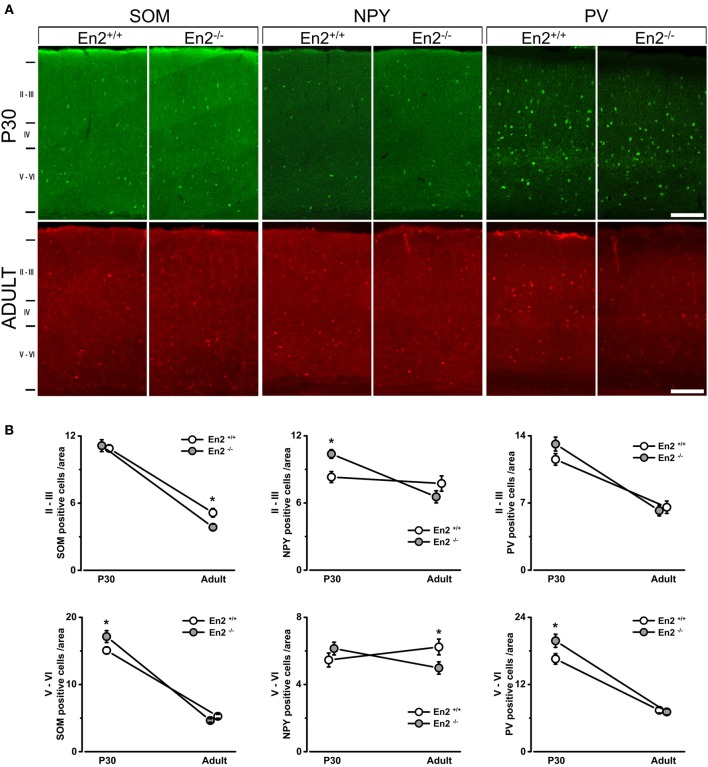
**Increased density of SOM^+^, NPY^+^, and PV^+^ cells in the primary visual cortex of juvenile *En2*^−/−^ mice**. **(A)** Representative SOM, NPY, and PV immunostainings taken at the level of the primary visual cortex in P30 and adult *En2*^+/+^ (white) and *En2*^−/−^ (gray) mice. **(B)** Quantification of immunohistochemistry experiments. Cell densities in layers II–III and V–VI are plotted as the mean number (± s.e.m.) of cell per area (see Materials and Methods). Note the increased density of SOM^+^ (layers V–VI), NPY^+^ (layers II–III), and PV^+^ (all layers) cells in *En2*^−/−^ mice at P30. A lower density of SOM^+^ cells was detected in the *En2*^−/−^ visual cortex at adult age. Statistical significance: ^*^p < 0.05 (*En2*^+/+^ vs. *En2*^−/−^). Scale bar = 100 μm.

Immunohistochemistry experiments were then performed to quantify SOM^+^, NPY^+^, and PV^+^ cell densities in the primary visual cortex of P30 and adult *En2*^+/+^ and *En2*^−/−^ mice. Different layers were identified by NeuN immunostainings in adjacent sections (Figure 2A). Immunostainings (Figure 3A) revealed a significantly higher number of SOM^+^ and PV^+^ interneurons throughout superficial (II–III) and deep (V–VI) cortical layers in both genotypes at P30, as compared to the adult age (SOM^+^: −53 and −65% in *En2*^+/+^, −65 and −73% in *En2*^−/−^; PV^+^: −43 and −55% in *En2*^+/+^, −53 and −64% in *En2*^−/−^; Two-Way ANOVA followed by Tukey test, *p* < 0.001 for all comparisons, *n* = 3 per genotype and age group; Figure 3B). Significant differences in SOM^+^, NPY^+^, and PV^+^ cell densities were detected between *En2*^+/+^ and *En2*^−/−^ mice, both at P30 and adult age. At P30, we found a significantly higher number of NPY^+^ (+21% in layers II–III), SOM^+^ (+14% in layers V–VI), and PV^+^ (+20% in layers V–VI) cells in the visual cortex of *En2*^−/−^ mice, as compared to *En2*^+/+^ controls (NPY, *p* = 0.017, Two-Way ANOVA followed by multiple *t*-test; SOM, *p* = 0.020 and PV, *p* = 0.030, Two-Way ANOVA followed by Tukey test, *n* = 3 per genotype; Figure 3B). In adult animals, *En2*^−/−^ mice showed a lower number (−25%) of SOM^+^ cells in layers II–III, and a lower number (−20%) of NPY^+^ cells in layers V–VI, as compared to age-matched controls (SOM, *p* = 0.024, Two-Way ANOVA followed by Tukey test; NPY, *p* = 0.040, Two-Way ANOVA followed by multiple *t*-test; *n* = 3 per genotype; Figure 3B). Table 2 summarizes the significant differences in glutamatergic and GABAergic markers observed between the *En2*^+/+^ and *En2*^−/−^ visual cortices at P30 and adult age.

### *En2*^−/−^ mice display normal overall development of visual function but enhanced binocularity of cortical neurons

**Figure 4:**
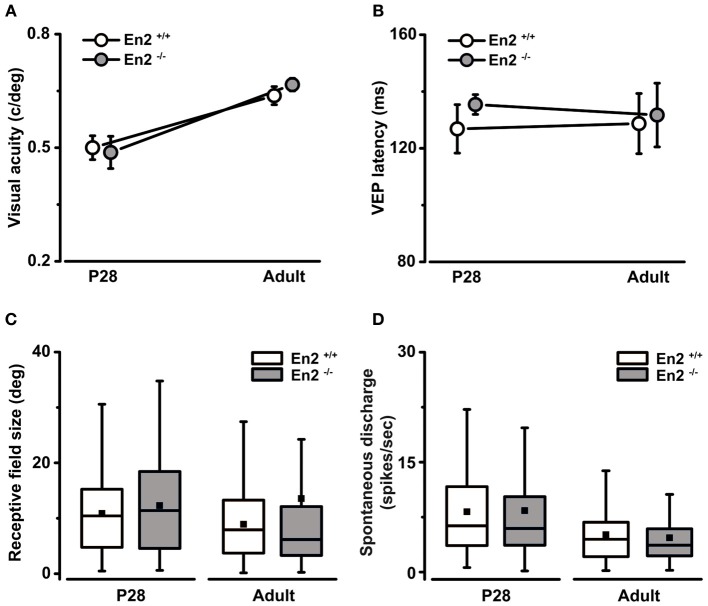
**Normal development of basic physiological parameters in the primary visual cortex of *En2*^−/−^ mice. (A,B)** *In vivo* electrophysiological VEP recordings from the primary visual cortex at P28 and adult ages revealed that the maturation of basal visual properties (visual acuity, **A**) and VEP latency of binocular responses **(B)** were not affected in *En2*^−/−^ mice. The circles depict mean ± s.e.m.; *En2*^+/+^, white; *En2*^−/−^, gray. **(C,D)** Analysis of single-unit recordings from visual cortical neurons showed no significant difference between *En2*^+/+^ (white) and *En2*^−/−^ (gray) mice in terms of receptive field size **(C)** and spontaneous activity **(D)** at either age. Data are summarized by box charts. Horizontal lines in the box chart denote the 25th, 50th, and 75th percentile values, the error bars denote the 5th and 95th percentile values while the square indicates the mean of the data.

We next investigated the functional consequences of the altered interneuron number in the *En2*^−/−^ visual cortex. We performed VEP and single unit recordings to measure basic physiological parameters in primary visual cortex of *En2*^+/+^ and *En2*^−/−^ mice at two different stages of postnatal development. Specifically, recordings were performed in juvenile (P28, peak of the critical period; *n* = 6 per genotype) and adult animals (P90–P120; *n* = 4 per genotype). First, by means of VEP recordings, we found that visual acuity developed normally in *En2*^−/−^ mice. Indeed, both *En2*^+/+^ and *En2*^−/−^ animals showed comparable acuity values at P28, and their spatial resolution increased similarly in adulthood (P28 vs. adult, Two-Way ANOVA followed by Tukey test, *p* < 0.05 for both genotypes; Figure 4A). Next, we analyzed latency of binocular VEP responses and we found neither genotype- nor age-dependent changes in this parameter (ANOVA, *p* = 0.86; Figure 4B). The absolute amplitude of binocular VEP responses was also comparable between genotypes (ANOVA, *p* = 0.3; data not shown).

Extracellular recordings of single-unit firing in response to visual stimulation with drifting light bar allowed us to measure the receptive field (RF) size of visual cortical neurons. We found that both *En2*^+/+^ and *En2*^−/−^ mice showed a tendency toward reduction of RF size in adulthood as compared to P28 (P28 vs. adult, Mann–Whitney test, *p* = 0.05 for *En2*^+/+^, *p* = 0.06 for *En2*^−/−^; Figure 4C). These data are consistent with the VEP findings, as cortical maturation is normally characterized by an age-dependent reduction of RF size that correlates with the acquisition of higher acuity values (Fagiolini et al., 1994; Gianfranceschi et al., 2003). We also evaluated spontaneous discharge of visual cortical neurons and we found lower spontaneous activity in adults as compared to juvenile animals, but no significant differences between *En2*^+/+^ and *En2*^−/−^ mice (P28 vs. adult, ANOVA followed by Dunn's test, *p* < 0.05; *En2*^+/+^ vs. *En2*^−/−^, *p* > 0.05; Figure 4D). Thus, a number of baseline physiological parameters appear to develop normally in *En2*^−/−^ mice.

**Figure 5:**
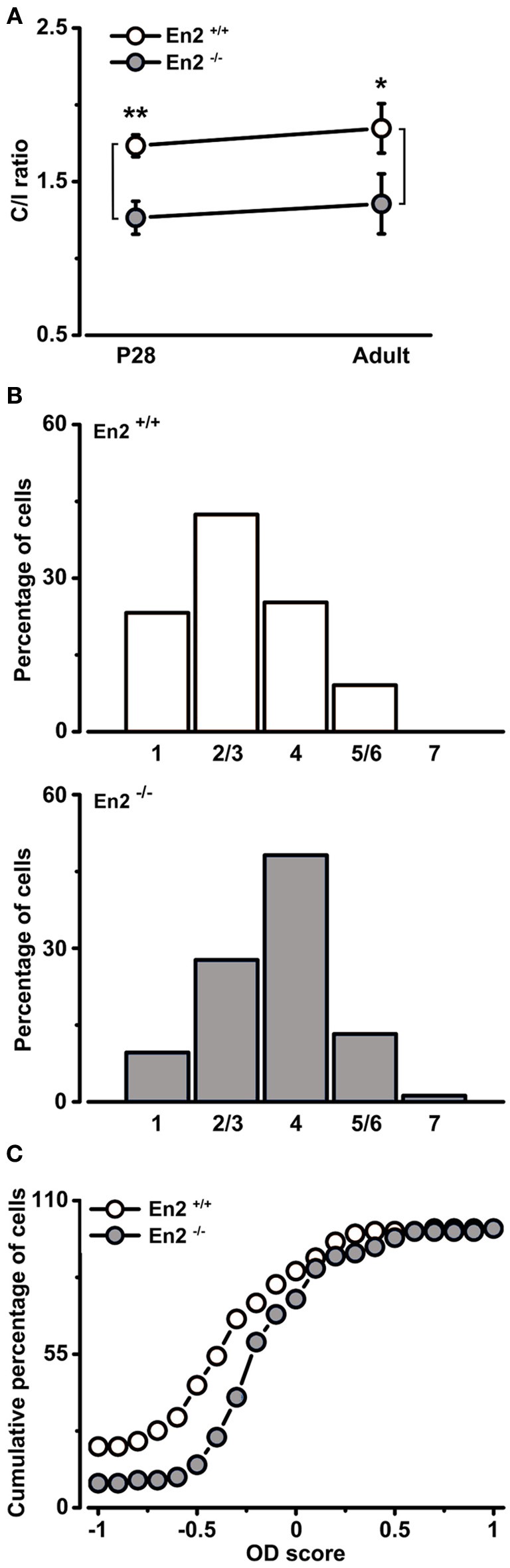
**Altered baseline binocularity in primary visual cortex of *En2*^−/−^ mice**. **(A)** Baseline binocularity was assessed by measuring the C/I VEP ratio at two different ages, P28 and adult. *En2*^−/−^ mice (gray) displayed a lower C/I ratio in comparison to *En2*^+/+^ animals (white) at both young and adult ages. The circles depict mean ± s.e.m. **(B)** OD distribution of *En2*^+/+^ (top, white) and *En2*^−/−^ (bottom, gray) adult mice. Note the higher proportion of class 4 cells of the OD histogram from *En2*^−/−^ animals (χ^2^ Test, *p* = 0.003). **(C)** Cumulative distribution of the OD score in *En2*^+/+^ (white) and *En2*^−/−^ (gray) adult mice. The two groups are significantly different from each other (see text for details). Statistical significance: ^*^p < 0.05, ^**^p < 0.01.

In contrast, analysis of how inputs from the two eyes integrate in visual cortical neurons revealed significant alterations in *En2*^−/−^ mice. First, we evaluated binocularity by measuring the contralateral-to-ipsilateral (C/I) VEP ratio (i.e., the ratio of VEP amplitudes recorded by stimulating the contralateral or ipsilateral eye). We found that *En2*^−/−^ mice displayed lower C/I ratios than *En2*^+/+^ both at P28 (*En2*^+/+^, 1.73 ± 0.07; *En2*^−/−^, 1.26 ± 0.11, *n* = 6 per genotype) and in adulthood (*En2*^+/+^, 1.85 ± 0.16; *En2*^−/−^, 1.36 ± 0.19, *n* = 4 per genotype; Two-Way ANOVA followed by Tukey test, *En2*^+/+^ vs. *En2*^−/−^; P28, *p* = 0.002; adult, *p* = 0.02; Figure 5A). Thus, while *En2*^+/+^ animals show a normal bias of cortical responses toward the contralateral eye, in the primary visual cortex of *En2*^−/−^ mice inputs from the two eyes are more balanced.

To further investigate baseline binocularity in adult animals, we performed extracellular recordings of single-unit firing in response to stimulation of each eye with a light bar drifting in the receptive field. OD was quantitatively assigned to each unit according to a five-point scale (Restani et al., 2009). As expected, the OD distribution of *En2*^+/+^ mice was biased toward the contralateral eye (Figure 5B, top). Conversely, the majority of the neurons in *En2*^−/−^ mice were equally driven by both eyes, resulting in a predominance (48.2%) of class 4 cells (Figure 5B, bottom). The two OD histograms of *En2*^+/+^ and *En2*^−/−^ mice were statistically different (χ^2^ Test, *p* = 0.003). We also calculated an OD score for each recorded neuron (Rittenhouse et al., 1999; Cerri et al., 2011). This analysis confirmed that the OD distribution recorded in *En2*^−/−^ animals was significantly different from that of *En2*^+/+^ mice (Kolmogorov–Smirnov Test, *p* < 0.001; Figure 5C).

### Normal retino-geniculate projections in *En2*^−/−^ mice

**Figure 6:**
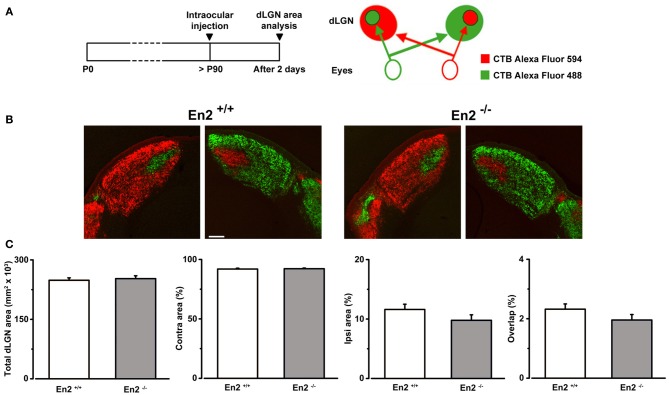
**Normal segregation of eye specific inputs in the dLGN of *En2*^−/−^ adult mice. (A)** Schematics of the experimental protocol for anterograde labeling of retino-geniculate afferents. **(B)** Representative coronal sections of both right and left dLGN in *En2*^+/+^ and *En2*^−/−^ mice. Scale bar: 200 μm. **(C)** Quantification of the total dLGN area, relative contralateral (contra) and ipsilateral (ipsi) areas, and percentage of overlap between inputs from the two eyes in the dLGN of in *En2*^+/+^ (white) and *En2*^−/−^ (gray) mice. No significant difference between genotypes is detectable in any of the parameters. Histograms depict mean ± s.e.m.

To exclude the possibility that the higher binocularity in *En2*^−/−^ mice was due to anatomical developmental defects in the visual pathway elicited by the loss of *En2*, we investigated segregation of eye-specific inputs in the dLGN in *En2*^+/+^ and *En2*^−/−^ animals. To visualize retino-thalamic afferents from both eyes, we performed intravitreal injections of CTB subunit fragment conjugates of different fluorescent dyes in adult mice of both genotypes. Fiber segregation was analyzed in coronal dLGN sections 2 days after injection (Figures 6A,B). We found a clear segregation of fibers from the two eyes in both *En2*^+/+^ and *En2*^−/−^ mice. Specifically, the total dLGN area, and the areas occupied by contralateral and ipsilateral eye inputs were superimposable between groups (Figure 6C; *t*-test, *p* > 0.25 for all comparisons; *En2*^+/+^, *n* = 5 and *En2*^−/−^, *n* = 3). Importantly, overlap of retinal projections from the two eyes was low (about 2%) in both genotypes, indicating a normal refinement of eye-specific inputs in the dLGN.

### *En2*^−/−^ mice are insensitive to a brief period of monocular deprivation

**Figure 7:**
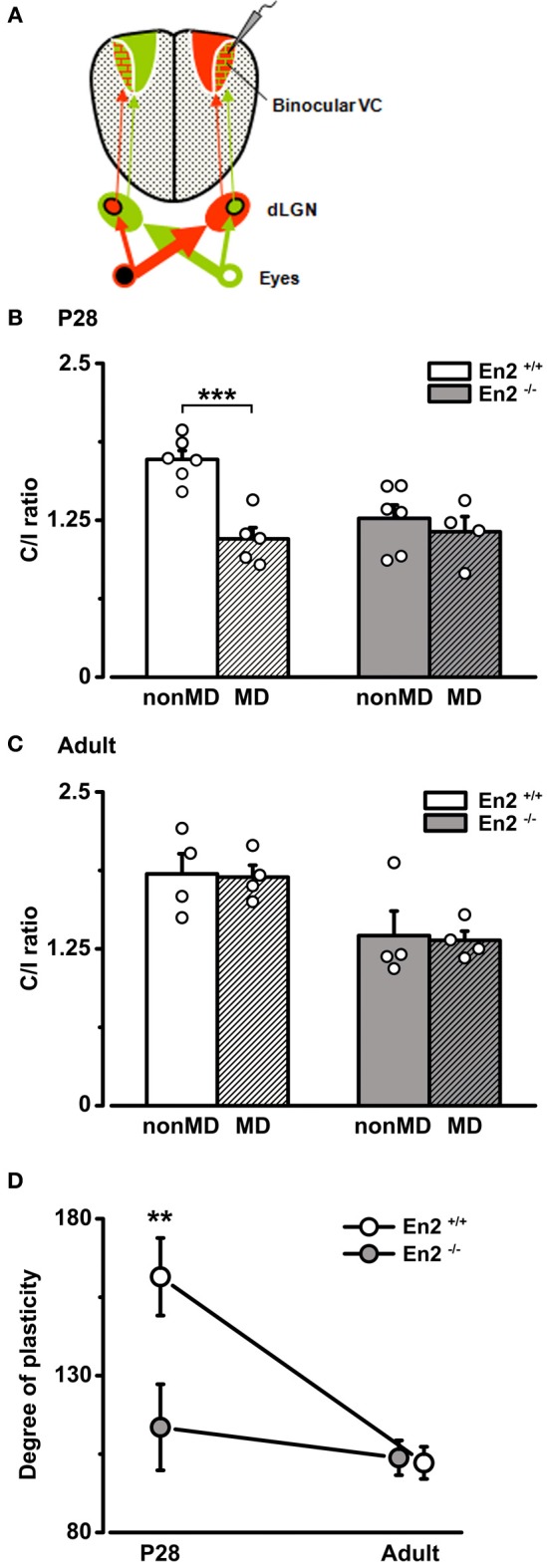
**Absence of OD plasticity in *En2*^−/−^ mice**. **(A)** Schematics of the experimental protocol for VEP recordings performed contralateral to the deprived eye. **(B,C)** C/I VEP ratios in *En2*^+/+^ (white) and *En2*^−/−^ (gray) mice either non-deprived (non-MD) or monocularly deprived for 3 days (MD; shaded columns). **(B)** Juvenile animals. Note the significant decrease in C/I ratio in *En2*^+/+^, while *En2*^−/−^ mice are not sensitive to brief MD. **(C)** Adult animals. No significant OD shift is measurable in either *En2*^+/+^ or *En2*^−/−^ mice after brief MD. Histograms depict mean ± s.e.m., whereas circles represent data from single animals. **(D)** Average level of OD plasticity ± s.e.m. for each age/genotype. The degree of plasticity was quantified for each monocularly deprived animal, as described in the Materials and Methods section. Note the significant OD plasticity in P28 *En2*^+/+^ but not *En2*^−/−^ mice. Statistical significance: ^**^p < 0.01, ^***^p < 0.001.

To determine how the loss of *En2* protein affects the susceptibility of visual cortical networks to activity-dependent modifications, we used a classical paradigm of experience-dependent plasticity (i.e., a brief period of 3 days of monocular deprivation, MD) in *En2*^+/+^ and *En2*^−/−^ mice. When performed during the critical period, 3 days of MD shift OD of cortical neurons toward the non-deprived eye (Levelt and Hübener, 2012). We performed the MD experiments at P28 which is the peak of plasticity, and at P90–P120, an age at which mice do not display OD changes following 3 days MD (Sawtell et al., 2003; Lehmann and Löwel, 2008; Sato and Stryker, 2008). To quantify the OD shift, we performed electrophysiological recordings in the hemisphere contralateral to the deprived eye (Figure 7A) and measured the C/I VEP ratio. We found that 3 days of MD at P28 produced a dramatic decrease of C/I ratio in the *En2*^+/+^ mice (*n* = 5; C/I ratio = 1.1 ± 0.09 vs. 1.73 ± 0.07 in non-MD animals), consistent with a loss of responsiveness of the deprived contralateral eye (Two-Way ANOVA followed by Tukey test, *p* < 0.001; Figure 7B). Conversely, juvenile *En2*^−/−^ mice showed no change in eye preference after MD (*n* = 4; C/I ratio = 1.16 ± 0.12 vs. 1.26 ± 0.11 in non-MD animals; Two-Way ANOVA followed by Tukey test, *p* > 0.05; Figure 7B). When MD was performed in adult age, neither *En2*^+/+^ (*n* = 4; C/I ratio = 1.82 ± 0.09 vs. 1.85 ± 0.16 in non-MD conditions) nor *En2*^−/−^ mice (*n* = 4; C/I ratio = 1.32 ± 0.07 vs. 1.36 ± 0.19 in non-MD conditions) displayed significant OD plasticity (non-MD vs. MD, Two-Way ANOVA followed by Tukey test, *p* > 0.05; Figure 7C).

The effects of MD were quantified in each group by an index (degree of plasticity) that increases with the magnitude of the OD shift. Plotting this index as a function of age revealed that *En2*^+/+^ mice, as expected, were sensitive to experience only in juvenile age (Two-Way ANOVA followed by Tukey test, *p* < 0.01; Figure 7D). In contrast, MD-induced plasticity was virtually absent in *En2*^−/−^ mice at both ages tested (Two-Way ANOVA followed by Tukey test, *p* > 0.05; Figure 7D). To evaluate possible effects of MD on the temporal characteristics of the response, we also calculated the onset latency of the VEP in deprived and naïve animals (P28). We found that onset VEP latency was in the order of 70 ms, with no significant differences between *En2*^+/+^ and *En2*^−/−^ mice, both in naïve conditions (non-MD, *n* = 6 per genotype) and after MD (*En2*^+/+^, *n* = 5; *En2*^−/−^, *n* = 4; One-Way ANOVA, *p* = 0.24; see Supplementary Material).

**Figure 8:**
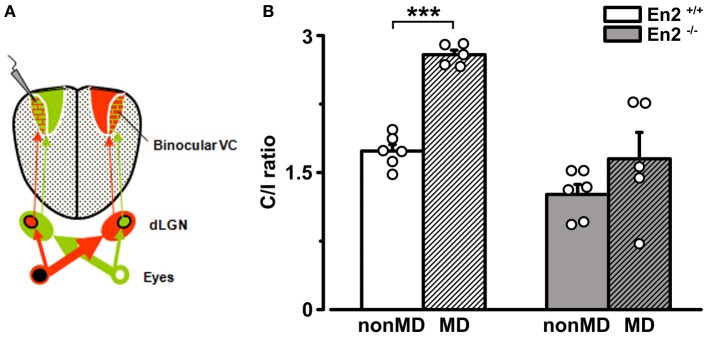
**Juvenile *En2*^−/−^ mice do not exhibit OD plasticity after brief MD in the hemisphere ipsilateral to the deprived eye. (A)** Schematics of the experimental protocol for VEP recordings performed ipsilateral to the deprivation. **(B)** C/I VEP ratios in *En2*^+/+^ (white) and *En2*^−/−^ (gray) mice either non-MD or monocularly deprived for 3 days (MD; shaded columns). Note the significant increase of C/I ratio in *En2*^+/+^, while *En2*^−/−^ animals do not display a consistent OD shift. Histograms depict mean ± s.e.m., whereas circles represent data from single animals. Statistical significance: ^***^p < 0.001.

It might be argued that the greater baseline binocularity of *En2*^−/−^ mice masks OD plasticity in the hemisphere contralateral to the deprived eye due to a “floor” effect, i.e., to the occlusion of a further decrease of the C/I ratio despite an unbalanced visual drive. To rule out this possibility, we performed recordings at P28–P30 in the visual cortex ipsilateral to the deprivation, where an increase in the C/I ratio should occur after MD (Figure 8A). Indeed, a further enhanced dominance of the contralateral, open eye was detected in MD *En2*^+/+^ mice (*n* = 5; C/I ratio = 2.79 ± 0.05 vs. 1.73 ± 0.07 in non-MD animals; Two-Way ANOVA followed by Tukey test, *p* = 0.0002; Figure 8B). Conversely, the C/I ratio of MD *En2*^−/−^ mice remained similar to that of naïve, undeprived mice (*n* = 5; 1.65 ± 0.29 vs. 1.26 ± 0.11 in non-MD animals; ANOVA followed by Tukey test, *p* > 0.05; Figure 8B). These data confirm the lack of experience-dependent changes in eye preference in the visual cortex of *En2*^−/−^ mice.

## Discussion

In this study, we exploited the well-known anatomical and functional features of the mouse visual system to investigate the relationship between GABAergic dysfunction and maturation of cortical function in *En2*^−/−^ mice, a model of ASD. In agreement with our previous study on the somatosensory cortex (Sgadò et al., 2013a), *En2*^−/−^ mice showed a reduced number of SOM^+^ and NPY^+^ interneurons in the adult (P90–P120) primary visual cortex, as compared to age-matched *En2*^+/+^ controls. However, when analyzed at P30 (during the critical period of visual cortical plasticity), *En2*^−/−^ mice showed an *increased* number of PV^+^, SOM^+^, and NPY^+^ interneurons in the visual cortex. These anatomical abnormalities were accompanied by a significant increase of binocularity in P28 and adult *En2*^−/−^ mice, and by the inability to respond to a brief monocular deprivation performed during the critical period and in adulthood.

### Altered expression of GABAergic markers in the visual cortex of *En2*^−/−^ mice

Higher mRNA levels of inhibitory markers and increased density of PV^+^, SOM^+^, and NPY^+^ interneurons were detected in the visual cortex of *En2*^−/−^ mice at P30, as compared to age-matched controls (Figures 1, 2, Table 2). This might suggest that an increased inhibitory tone is present in the *En2*^−/−^ visual cortex during the critical period for visual cortical plasticity.

This might be due to the loss of *En2* transcriptional regulation of the expression of GABAergic genes. Several recent studies addressed the physiological properties of cortical inhibitory interneurons, namely PV^+^ and SOM^+^ cells; little is known about the activity of NPY^+^ cells. PV^+^ and SOM^+^ cells target distinct compartments on pyramidal cells, PV^+^ cells providing perisomatic inhibition, and SOM^+^ cells forming synapses onto dendrites (Markram et al., 2004; Ascoli et al., 2008). It is well-established that perisomatic inhibition by PV^+^ cells contributes to OD plasticity in the visual cortex (Di Cristo et al., 2004). However, recent studies suggest that SOM^+^ cells might also play a role, as both cell types show extensive physiological maturation from eye opening to the peak of OD plasticity (Lazarus and Huang, 2011). However, these two cell types present profound differences in terms of stimulus sensitivity and inhibitory activity. As compared to PV^+^ cells, SOM^+^ cells provide a weaker but selective inhibition in response to visual stimuli (Ma et al., 2010; Wilson et al., 2012). PV^+^ and SOM^+^ cells are also different in the way they inhibit other interneuron subtypes: PV^+^ cells inhibit one another but not other interneurons, while SOM^+^ cells inhibit all other interneuron subtypes but not one another (Pfeffer et al., 2013). Thus, an increased number of SOM^+^ and PV^+^ interneurons during the critical period might have a profound impact on interneuron network activity and visual cortical physiology in *En2*^−/−^ mice (see also the paragraphs below).

Conversely, a lower number of SOM^+^ and NPY^+^ (but not PV^+^) cells was detected in the adult visual cortex of *En2*^−/−^ mice, as compared to age-matched controls (Figure 3 and Table 2). These results suggest that the transcription factor *En2* may have a direct effect on specific interneuron populations by modifying the expression of GABAergic-specific genes; our recent transcriptome study, performed in the hippocampus, supports this hypothesis (Sgadò et al., 2013b). The present results are in good agreement with our previous study, showing a reduction of SOM^+^, NPY^+^, and PV^+^ cells in the somatosensory cortex of adult *En2*^−/−^ mice (Sgadò et al., 2013a). However, an important difference emerges from these two studies. Specifically, the PV^+^ cell population seems less affected in the visual cortex as compared to the somatosensory cortex, suggesting that different sensory cortices display a distinct profile of interneuron maturation in *En2*^−/−^ mice. Indeed, the somatosensory cortex has a greater number of PV^+^ interneurons as compared to the visual cortex (Tanahira et al., 2009). Our data also showed a developmental decrease of SOM^+^ and PV^+^ cells in the visual cortex of *En2*^+/+^ mice, though less pronounced than that observed in *En2*^−/−^ animals (Figure 3B). Indeed, a decrease of inhibitory interneurons in the mouse frontal cortex has been described to occur during postnatal development (Eto et al., 2010). In addition, a recent study showed that in layers II–III of the mouse primary visual cortex, PV^+^ cell density increases from P28 to P42, and then decreases until P70 (Ye and Miao, 2013), with no change of total cell density. Accordingly, we observed a decrease of PV^+^ cell density between P30 and P90–P120 in both genotypes, with no change in the density of total neuron numbers. This decrease was generally more pronounced in *En2*^−/−^ mice, suggesting that *En2* loss may impact the slow postnatal maturation process of specific interneuron subtypes, as discussed above. However, it should be pointed out that another study did not reveal a developmental decrease of PV^+^ and SOM^+^ cell densities in the mouse visual cortex (Gonchar et al., 2007). The different mouse genetic background and counting procedures used in our study might account for this discrepancy.

### Role of *En2* in the mouse visual system

We found no alterations in the retino-geniculate pathway in *En2*^−/−^ mice, which showed a normal segregation of eye-specific inputs in the dLGN (Figure 5). In Xenopus and chick embryos, En proteins have an instructive role in guiding retinal axons to the optic tectum (Brunet et al., 2005; Wizenmann et al., 2009). In the present study, we did not examine early stages of retinogeniculate axon guidance but we chose to concentrate our attention on eye-specific segregation as a possible neuroanatomical basis for the altered binocularity in *En2*^−/−^ mice. Contrary to what previously reported (Brielmaier et al., 2012), *En2* mRNA is expressed in the mouse visual cortex during postnatal development (Figure 1), confirming our previous data showing the expression of En1/2 protein throughout all cortical areas (Sgadò et al., 2013a). Thus, in the mouse visual system, *En2* might have a peculiar role on the maturation of specific interneuron populations. A similar role has been described for the homeobox-containing transcription factor Otx2, which exerts its effects on PV^+^ cell maturation in a trans-synaptic manner (Sugiyama et al., 2008). Like Otx2, *En2* is secreted onto target cells (Fuchs et al., 2012); it would be therefore interesting to investigate whether a secretion-dependent mechanism underlies the effect of *En2* onto cortical interneuron maturation during postnatal development.

### Normal visual physiological parameters but increased binocularity in *En2*^−/−^ mice

Our electrophysiological analysis demonstrated that several basic physiological properties (visual acuity, latency and amplitude of the visual response, receptive field size, spontaneous discharge) were within the normal range in both juvenile and adult *En2*^−/−^ animals. Therefore, deletion of *En2* has no impact on these parameters but clearly affects binocularity in both juvenile and adult ages (Figure 5). VEP and single unit recordings showed indeed a more balanced C/I ratio and a higher proportion of class 4 neurons (i.e., cells equally responsive to stimulation of the contralateral and ipsilateral eye) in *En2*^−/−^ mice. A higher proportion of binocular cells is typically found in the visual cortex of immature rodents and a contralateral bias is established as development proceeds (Fagiolini et al., 1994). Manipulations such as dark rearing (Fagiolini et al., 1994; McCurry et al., 2010) or deletion of the transcription factor AP2γ (Pinto et al., 2009) impair maturation of the visual cortex and lead to the maintenance of a higher proportion of binocular units. However, dark rearing and knockout of AP2γ are also characterized by altered development of visual acuity, receptive field size, spontaneous discharge, and latency of visual drive (Fagiolini et al., 1994; Pinto et al., 2009), parameters that were found to be within the normal range in *En2*^−/−^ animals. Thus, lack of *En2* selectively affects balance of eye-specific drive in the primary visual cortex while leaving the development of other functional properties unaffected. A similar phenotype was reported in mice lacking the immediate-early gene Arc, that display normal map organization and visual acuity but enhanced baseline binocularity (McCurry et al., 2010). Altogether these experiments demonstrated that the molecular pathways that generate individual sensory response properties are separable in cortical networks (Fagiolini et al., 2003).

The enhanced binocularity of *En2*^−/−^ mice is likely of cortical origin and not secondary to alterations in retinogeniculate afferents, as a normal segregation of eye-specific inputs was found in the dLGN (Figure 6). It is unlikely that the higher C/I ratio is directly linked to the alterations of GABAergic markers that we documented at the molecular and neuroanatomical level. Indeed, an increased binocularity was evident in both juvenile and adult *En2*^−/−^ mice, ages at which GABAergic markers were up- and down-regulated, respectively. Another possibility is that modifications of callosal connections account for this phenotype of *En2*^−/−^ animals. Indeed, we and others (Restani et al., 2009; Cerri et al., 2010; Pietrasanta et al., 2012; Zhao et al., 2013) have recently shown that callosal afferents contribute to binocularity in rodents by carrying inputs from the ipsilateral eye to cortical neurons. Along this line, the altered binocularity of mice lacking *En2* could be explained by a functional “hyperconnectivity” of transcallosal pathways.

### MD fails to elicit eye preference changes in *En2*^−/−^ mice

MD is a classical experimental paradigm to evaluate the susceptibility of cortical networks to undergo experience-dependent modifications, and to investigate the regulation of critical period timing. In mice there is an age-dependent decline in the potential for OD plasticity (Lehmann and Löwel, 2008), so that 7 days of MD are effective in changing eye preference in young adult mice (P90–P120) but completely ineffective in older animals (>P200). Adult OD plasticity can be measured by different techniques, such as VEPs (Sawtell et al., 2003) and optical imaging of intrinsic signals (Hofer et al., 2006; Lehmann and Löwel, 2008). The developmental trajectory of OD plasticity can be significantly impacted by rearing conditions; for example environmental enrichment significantly extends OD plasticity into late adulthood (Baroncelli et al., 2010; Greifzu et al., 2014). In this study, we adopted a short period of MD (3 days) that induces a change in eye preference during the critical period but is ineffective in adults (Sawtell et al., 2003; Lehmann and Löwel, 2008; Sato and Stryker, 2008).

Application of a brief period of monocular occlusion in juvenile mice lacking *En2* failed to produce an OD shift while it was extremely effective in P28 *En2*^+/+^ animals. Response to MD was also not detectable in adult *En2*^−/−^ mice. Given the lower baseline C/I ratio of *En2*^−/−^ mice, we wondered whether the lack of OD plasticity in these animals may be ascribable to a “floor” effect, i.e., to the occlusion of a further decrease of the C/I ratio in the hemisphere contralateral to eyelid suture despite an unbalanced visual drive. To address this issue, we performed experiments in the visual cortex ipsilateral to the deprivation (McCurry et al., 2010) and we were able to demonstrate a genuine deficit in plasticity. Thus, the lack of *En2* impairs activity-dependent modifications at the peak of the “normal” critical period.

The alterations in the inhibitory cortical circuits of *En2*^−/−^ mice may impact directly on susceptibility to MD in these animals. Hyperpolarizing GABA neurotransmission is indeed a well-known regulator of critical period plasticity (Levelt and Hübener, 2012). For example, animals lacking the GABA biosynthetic enzyme GAD65 are completely resistant to MD and display experience-dependent plasticity only following enhancement of GABA-mediated transmission with benzodiazepines (Hensch et al., 1998; Fagiolini and Hensch, 2000). On the other hand, acceleration of GABAergic circuit maturation by BDNF overexpression leads to a precocious closure of the critical period (Huang et al., 1999). These experiments have led to the idea that, during maturation of GABAergic system, a first threshold of inhibition allows plasticity to occur, while the reaching of a second inhibitory threshold signals the closure of the sensitive period (Hensch, 2005). In keeping with this hypothesis, reduction of GABAergic inhibition in adulthood by different manipulations reinstates susceptibility to MD (Sale et al., 2007; Harauzov et al., 2010; Greifzu et al., 2014). In *En2*^−/−^ animals, several markers of inhibition were significantly upregulated at P30 as compared to *En2*^+/+^ mice (Figures 1, 3). Thus, a possibility for the absence of OD plasticity in these animals is that a precocious development of the inhibitory system may trigger the closure of the sensitive period for experience-dependent modifications. This hypothesis remains to be tested with MD experiments in younger (e.g., P20) *En2*^−/−^ animals. A premature peak of plasticity coupled with an accelerated maturation of GABAergic markers has been described in mice overexpressing BDNF (Huang et al., 1999).

Our findings demonstrate that the lack of *En2* affects susceptibility to MD but not visual functions. To our knowledge, this is one of the few examples in which two normally strongly intermingled processes (maturation of visual acuity and OD plasticity) may be regulated independently. Interference with *En2* function allowed us to dissect a pathway that influences experience-dependent modifications but not visual performance.

### Cortical plasticity deficits in mouse ASD models

Susceptibility of cortical networks to visual deprivation has been previously examined in other murine models of ASD. Analysis of mice lacking the transcription factor *Mecp2* (a model for Rett syndrome) revealed alterations in cortical plasticity (Tropea et al., 2009b). In another animal model of autism (mice deficient for Ube3a, a ligase implicated in Angelman syndrome), OD plasticity following brief MD is severely impaired (Yashiro et al., 2009). This is in keeping with the present data reporting the absence of MD-induced changes in eye preference in the *En2* mouse model of autism. These data suggest that the alteration of plasticity mechanisms might underlie aberrant development of cortical circuits and the associated behavioral deficits in ASD.

## Author contributions

Manuela Allegra and Sacha Genovesi designed and performed experiments, analyzed data and wrote the paper. Marika Maggia, Maria C. Cenni, Giulia Zunino and Paola Sgadò performed experiments. Matteo Caleo and Yuri Bozzi provided funding, conceived the study, analyzed data and wrote the paper.

### Conflict of interest statement

The authors declare that the research was conducted in the absence of any commercial or financial relationships that could be construed as a potential conflict of interest.
