# Supplementary material for: Altered GABAergic markers, increased binocularity and reduced plasticity in the visual cortex of Engrailed-2 knockout mice
Source: Front Cell Neurosci. 2014 Jun 17;8:163. doi: 10.3389/fncel.2014.00163 (PMC4060086; doi:10.3389/fncel.2014.00163)
Supplement: Supplementary file 1 [file Presentation1.PDF]

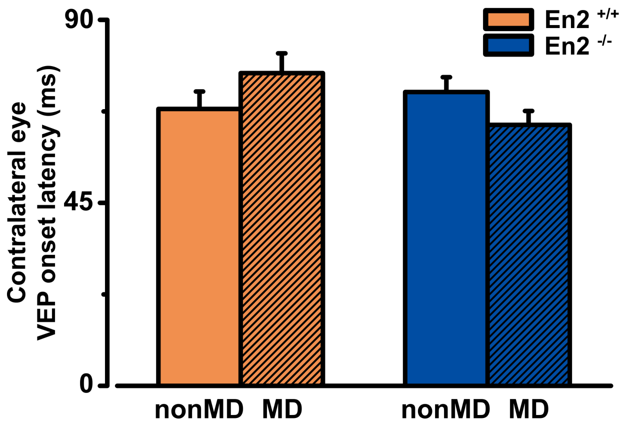

Supplementary Figure.

VEP onset latency in *En2*$^{+/+}$ (orange) and *En2*$^{-/-}$ (blue) deprived (MD) and nondeprived (nonMD) animals (P28). VEP onset latency was in the order of 70 ms, with no significant differences between *En2*$^{+/+}$ and *En2*$^{-/-}$ mice, both in nonMD conditions and after MD (one way ANOVA, $p = 0.24$). The number of animals used is as follows: nonMD, n = 6 per genotype; MD, n = 5 *En2*$^{+/+}$ and n = 4 *En2*$^{-/-}$.
